# *Candida glabrata*’s Genome Plasticity Confers a Unique Pattern of Expressed Cell Wall Proteins

**DOI:** 10.3390/jof4020067

**Published:** 2018-06-05

**Authors:** Eunice López-Fuentes, Guadalupe Gutiérrez-Escobedo, Bea Timmermans, Patrick Van Dijck, Alejandro De Las Peñas, Irene Castaño

**Affiliations:** 1 Instituto Potosino de Investigación Científica y Tecnológica (IPICYT), División de Biología Molecular, Camino a la Presa San José 2055, San Luis Potosí, SLP 78216, Mexico; eunice.lopez@ipicyt.edu.mx (E.L.-F.); maria.gutierrez@ipicyt.edu.mx (G.G.-E.); cano@ipicyt.edu.mx (A.D.L.P.); 2 KU Leuven, Laboratory of Molecular Cell Biology, Kasteelpark Arenberg 31 bus 2438, 3001 Leuven, Belgium; bea.timmermans@kuleuven.vib.be (B.T.); patrick.vandijck@kuleuven.vib.be (P.V.D.); 3 VIB-KU Leuven Center for Microbiology, 3001 Leuven, Belgium

**Keywords:** *Candida glabrata*, cell wall proteins, adherence, virulence, fluconazole resistance, genome plasticity, subtelomeric silencing, clinical isolates

## Abstract

*Candida glabrata* is the second most common cause of candidemia, and its ability to adhere to different host cell types, to microorganisms, and to medical devices are important virulence factors. Here, we consider three characteristics that confer extraordinary advantages to *C. glabrata* within the host. (1) *C. glabrata* has a large number of genes encoding for adhesins most of which are localized at subtelomeric regions. The number and sequence of these genes varies substantially depending on the strain, indicating that *C. glabrata* can tolerate high genomic plasticity; (2) The largest family of CWPs (cell wall proteins) is the EPA (epithelial adhesin) family of adhesins. Epa1 is the major adhesin and mediates adherence to epithelial, endothelial and immune cells. Several layers of regulation like subtelomeric silencing, *cis-*acting regulatory regions, activators, nutritional signaling, and stress conditions tightly regulate the expression of many adhesin-encoding genes in *C. glabrata*, while many others are not expressed. Importantly, there is a connection between acquired resistance to xenobiotics and increased adherence; (3) Other subfamilies of adhesins mediate adherence to *Candida albicans*, allowing *C. glabrata* to efficiently invade the oral epithelium and form robust biofilms. It is noteworthy that every *C. glabrata* strain analyzed presents a unique pattern of CWPs at the cell surface.

## 1. Introduction

Fungal invasive infections have become increasingly frequent in hospitals worldwide, in part, as a result of higher numbers of immunocompromised patients. Bloodstream infections are particularly dangerous since they are associated with high mortality rates [1]. In immunocompromised patients, *Candida* species are the most commonly isolated fungal species from bloodstream infections (candidemia) in the US, of which *Candida albicans* is the most frequently found species followed by *Candida glabrata* [2,3]. Candidemias have remained stable over the last decade but several studies report decreasing frequency of *C. albicans* and increases of other non-*albicans* species. Among these species, *C. glabrata* is of particular concern since it is innately less susceptible to azoles [4,5]. Alarmingly, some studies have found resistance to echinocandin class of antifungals among the already azole resistant *C. glabrata* clinical isolates in the US [6].

Adherence to different cells and surfaces is considered a crucial virulence factor in pathogens. In this regard, *C. glabrata* in part owes its success as a pathogen to the ability to adhere tightly to several types of mammalian host cells (epithelial, endothelial and immune cells), to other microbes (bacteria and other *Candida* species), and to medical devices and to form complex structures called biofilms. The frequency with which each of these species is isolated from patients, correlates with the number of genes encoding a specific class of Cell Wall Proteins (CWPs) called adhesins. The genome of *C. glabrata* contains an unusually large repertoire of cell wall protein-encoding genes, many of which are adhesin-like encoding genes. These proteins have the conserved structure of surface exposed CWPs (see below). In the *C. glabrata* reference genome there are 66 genes encoding adhesin-like proteins. The number of these genes and the CWPs actually displayed at the cell wall varies widely between clinical isolates of *C. glabrata*. Some of these clinical isolates contain up to 101 adhesin-encoding genes in their genomes [7,8,9,10]. 

In this review we will focus on the *EPA* (Epithelial adhesin) family of genes, emphasizing the large variability between different *C. glabrata* strains regarding the number of genes present per genome. We will describe the important role played by the major adhesins Epa1, Epa6, and Epa7 in mediating adhesion to host cell tissues, medical devices, and other microbes, and their unique expression pattern.

## 2. Role of Adherence during Infection

### 2.1. Adherence in Candida glabrata Is a Virulence Factor

Adherence to human host tissues and medical devices like catheters is thought to be the first step in the development of an infection caused by *C. glabrata*. This leads to colonization and invasion of the sterile tissues of the host. *C. glabrata* has been shown to adhere tightly to several types of mammalian cells such as, (1) epithelial cell lines derived from various organs (Lec2, HEp2, CHO, and HeLa among others) [11,12,13]; (2) coronary endothelium, endothelial cell lines as HBEC, HRGEC, and other endothelial cells [13,14,15]; (3) different types of immune cells like macrophages, natural killer, and dendritic cells [16,17,18]. *C. glabrata* also binds to some components of the host extracellular matrix like fibronectin (through an identified fibronectin receptor) [19] and laminin-332 [20,21]. In addition, *C. glabrata* can also form complex communities called biofilms that are adhered to different surfaces and embedded in an extracellular matrix. For example, *C. glabrata* can make biofilms on vascular and urinary catheters, cardiac devices like prosthetic valves and pacemakers (reviewed in [22]). The role of adhesins in the different stages of biofilm formation is discussed in another review in this volume [23]. The ability to adhere to different host tissues or surfaces allows *C. glabrata* cells to persist in a nourishing host niche, or to form protective biofilms to resist antifungal drugs. Thus, adherence is considered a very important virulence factor [24].

### 2.2. Adherence Is Mediated by Cwps in C. glabrata

The fungal cell wall is the first point of interaction with the environment. In *C. glabrata*, the cell wall contains a wide variety of so-called CWPs classified into two large groups based on type of bond with which they are associated to the cell wall: (a) Proteins linked through a mild alkali-sensitive linkage to β-1,3-glucan (Pir proteins), and (b) proteins containing glycosylphosphatidylinositol (GPI) modified anchor covalently bound to the β-1,6-glucan [25,26]. 

Pir (protein with internal repeats) proteins contain an N-terminal signal peptide for secretion, a Kex2 protease cleavage site, a central domain with a variable number of tandem repeats which are *O*-glycosylated, and a C-terminal region containing four cysteine residues [26,27]. GPI-modified CWPs comprise a large family of proteins exposed at the cell surface and covalently bound to the cell wall. Adherence of fungal cells is generally mediated by adhesins, which belong to the GPI-anchored class of proteins. Fungal adhesins consist of four general domains. (1) A signal peptide at the N-terminus for secretion; (2) The functional N-terminal domain is involved in ligand binding, which can recognize carbohydrates or protein ligands, depending on the particular adhesin; (3) The N-terminal domain is followed by a low complexity domain, which contains a variable number of repeated motifs of amino acids, which usually are rich in serine and threonine and is highly glycosylated; (4) The C-terminal end contains a GPI addition signal that is responsible for covalent crosslinking of the protein to the cell wall [13,28,29,30].

### 2.3. C. glabrata Shows High Variability in the Number and Sequence of CWP-Encoding Genes

Initial sequencing of the *C. glabrata* genome (strain CBS138 or ATTC 2001, here referred to as CBS138), in 2004 [31], revealed the presence of many putative CWP-encoding genes in comparison with the benign and closely related yeast *Saccharomyces cerevisiae* [10]. *C. glabrata* belongs to the *Nakaseomyces* genus, which comprises two subgroups. *C. glabrata* is part of the second subgroup (called the “*glabrata* group”) composed by *C. glabrata*, *N. delphensis* (a non-pathogenic species), and *C. bracarensis* and *C. nivariensis*. These last two species are closely related to *C. glabrata* and have been isolated from patients, albeit at a much lower frequency than *C. glabrata* [32]. The Epa family of adhesins encoded by the *EPA* genes confers this ability to adhere to epithelial and endothelial cells [11,13,33,34]. In fact, the capacity to colonize and produce disease in the human host coincides with the expansion of the *EPA* genes in *C. glabrata* compared to the nonpathogenic *N. delphensis*, *C. castellii*, and *N. bacillisporus* [32,35]. In different strains of *C. glabrata* isolated from patients worldwide there is an enormous variability in the number and type of adhesin-encoding genes. In some recently sequenced clinical isolates, about 1.3% of the genome encodes putative CWPs [7] and some other isolates contain 101 adhesin-like genes [10].

It is noteworthy that high levels of homologous, nonallelic recombination can account for some of the variability. The majority of these genes are located at subtelomeric regions where recombination can occur [24,36]. The genetic variation found between these isolates mainly affects CWPs. These genes accumulate many rearrangements such as deletions and duplications, thus behaving as recombinational hotspots [7].

### 2.4. Adherence to a Variety of Surfaces Is Mediated by the Presence of a Large Family of (GPI)-Modified Proteins at the Cell Surface

Based on a phylogenetic tree of the ligand binding N-terminal domain of adhesins, these can be divided into seven subgroups designated clusters I through VII. The *EPA* and the *PWP* genes conform two homogeneous subfamilies to which only *EPA* or *PWP* genes belong. The *AWP* (Adhesin-like wall protein) genes and other adhesin-like encoding genes belong to the 5 other different subfamilies [28]. There is little relationship between the N-terminal domains of clusters III–VII [25,28]. The *EPA* genes (cluster I) belong to the largest subgroup of adhesins with 18 genes in the sequenced strain CBS138 [31] and 23 in the laboratory standard strain BG2 [37]. Even among the orthologues of *EPA* genes between these two standard strains, there is high variability as the orthologues are not identical in sequence [28].

The Pwp family of adhesins (cluster II) is composed of at least seven members (Pwp1-7), and their N-terminal domain shares similarity to the corresponding domain of the Epa proteins, particularly in the conserved PA14 domain (Anthrax protective antigen) [28]. Pwp7 has been shown to mediate adherence to endothelial cells [14] and could also play a role in aggregation. 

Proteomic analysis of CWPs covalently bound to the cell wall of several *C. glabrata* strains identified six proteins of the Awp group: Awp1-6 [25,38]. A recent subsequent proteome analysis of highly hyperadherent clinical isolates discovered 6 novel adhesins of the Awp family: Awp8, Awp9, Awp10, Awp11, Awp12, and Awp13 [8]. 

Many of the adhesin-like CWPs of *C. glabrata*, including Epas, Awps, and Pwps, contain a variable number of repeated motifs of the VSHITT or SFFIT sequence. These repeats conform mega-satellites, which are unusually long tandem repeated sequences that are only present in *C. glabrata* [28,39]. The number of repeats in these mega-satellites has an impact on the length of the CWP and in some cases a larger number of repeats in the mini-satellites has been correlated with increased adherence, flocculation, or biofilm formation [40].

### 2.5. EPA Family

As stated above, the largest family of CWPs is the Epa family of adhesins. Most of the *EPA* genes are located in subtelomeric regions [33,34]. The first member discovered of this family was *EPA1*, which encodes for the major adhesin in vitro in *C. glabrata* (Epa1) [11]. Epa1 N-terminal, ligand-binding domain, shares the PA14 domain present in many carbohydrate-binding lectins. The role of Epa1 in vivo is also important, as several studies have found increased expression of *EPA1* [12,41,42], but other adhesins must also play important roles since *epa1*∆ strains display no defect in colonization phenotype in a mouse model of systemic infection [11]. Also, there are examples of highly adherent isolates that do not show high levels of *EPA1* expression [8,9,43].

Epa6 and Epa7 have also been shown to be functional adhesins involved in kidney and bladder colonization in vivo [33,44]. When Epa1, Epa6, and Epa7 are heterologously expressed in the non-adherent *S. cerevisiae*, they confer adherence to epithelial and endothelial cells in vitro and to other organisms as well [11,13,33]. Epa1, Epa6, and Epa7 bind carbohydrates containing a terminal galactose residue, although the specificity varies for these three adhesins [13]. The ligand binding specificity and the classification of Epa N-terminal subtypes is described in detail in [23] in this issue. 

## 3. Regulation of Expression of *EPA* Genes 

### 3.1. Subtelomeric Silencing and Cis-Acting Elements Regulate the Expression of EPA Genes

In general, adhesins are not constitutively expressed. It is possible that differentially regulated CWPs enable *C. glabrata* to change its adhesion capacity and adapt to the different abiotic surfaces, human tissues, or other microorganisms [8]. All the sequences adjacent to the telomeres of the sequenced CBS138 strain contain at least one putative adhesin-encoding gene [25]. The majority of the *EPA* genes are localized at these subtelomeric regions and this localization implies in some strains, like BG2, that the expression of the *EPA* genes is controlled by chromatin-based subtelomeric silencing [33,34]. Subtelomeric silencing is a regional and global transcriptional repression independent of the identity of the gene [45]. In *C. glabrata*, subtelomeric silencing requires the SIR complex (Sir2, Sir3, and Sir4), Rif1, Rap1, and yKu proteins (yKu70 and yKu80) and can extend >20 kb away from the telomere [34,44,46]. However, different telomeres in *C. glabrata* have different protein requirements for silencing. For instance, the proteins yKu70 and yKu80 are not required in the right telomere of the chromosome E (E_-R_) where the *EPA1* forms a cluster with *EPA2* and *EPA3* [46]. Furthermore, not all of the *C. glabrata* strains display an efficient negative regulation by subtelomeric silencing of the *EPA* genes as in the BG2 strain. Among a collection of 79 clinical isolates, 11 were found to be hyperadherent under conditions where most of the *EPA* genes are silenced in the BG2 strain. Analysis of the *SIR3* sequence of the hyperadherent isolates, revealed several polymorphisms that could account for a less efficient protein to establish or maintain subtelomeric silencing in the hyperadherent strains [12]. In this work, the CBS138 strain was found to be hyperadherent and the *SIR3* gene shares some of the polymorphisms identified in the hyperadherent clinical isolates [12].

In addition to the Chr E_-R_, silencing has been tested in other telomeres of *C. glabrata*. The *EPA4* and *EPA5* genes are located in the right telomere of chromosome I (Chr I_-R_). These genes form an almost perfect inverted repeat and subtelomeric silencing can propagate at least 24 kb. Silencing in this subtelomeric region depends on Rap1, Sir complex, Rif1, and yKu proteins [34,46]. In addition, silencing at both ends of chromosome C, where *EPA6* and *EPA7* are located, depends on the SIR complex and yKu proteins [33,46]. Therefore, at least in some *C. glabrata* strains, subtelomeric silencing is a global regulatory network which plays an important role in the expression pattern of the adhesins-encoding genes. 

In this regard, even though many *EPA* genes are silenced, their expression can be induced under different environmental conditions. This specific induction of *EPA* expression could have important implications in terms of adaptation to nutritional and oxidative stress or the presence of xenobiotics encountered in different host niches. Thus, *C. glabrata* cells could respond to changing environmental conditions by expressing a different set of CWPs. For example, in strain BG2, *EPA6* is induced during urinary tract infections due to low levels of nicotinic acid, a precursor of NAD^+^. The histone deacetylase Sir2, which is part of the SIR complex and essential for silencing of subtelomeric regions, requires NAD^+^ for its enzymatic activity. Since *C. glabrata* is an auxotroph of nicotinic acid, a milieu with low concentrations of NAD^+^ results in inactivation of Sir2, relief of silencing, and expression of *EPA6* [44]. *EPA2* expression is induced by the transcription factors Yap1 and Skn7 under conditions of oxidative stress [47]. *EPA1*, *EPA3*, *EPA7*, and *EPA22* are expressed during biofilm formation [38] and *EPA3* is also highly induced by osmotic stress and glucose starvation [48].

### 3.2. EPA1 Is Tightly Regulated by Cis-Acting Elements

**Figure 1:**
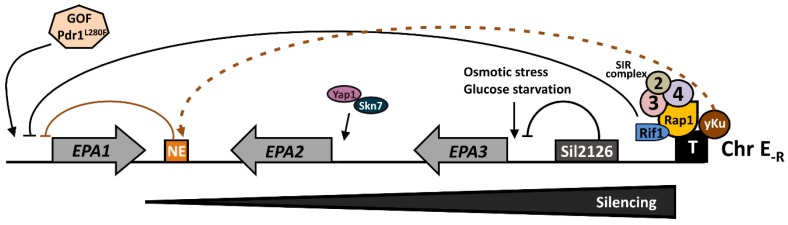
Map of the right subtelomeric region of chromosome E (E_-R_) where the *EPA1*, *EPA2*, and *EPA3* cluster is localized. *EPA1* expression is negatively regulated by the *cis*-acting element called Negative Element (NE, orange rectangle) shown as an orange T arrow, which depends on yKu70 and yKu80 proteins (yKu, brown circle). The dashed orange arrow indicates the genetic requirement of yKu for the activity of NE. *EPA2* is induced under oxidative stress and requires Yap1 (purple) and Skn7 (dark blue) transcription factors. *EPA3* is repressed by the protosilencer Sil2126 (dark gray rectangle), indicated by the black T arrow from Sil2126 to *EPA3* promoter. *EPA1*, *EPA2*, and *EPA3* are subject to another layer of global regulation called subtelomeric silencing (indicated by a horizontal black triangle below the chromosome and a black T arrow from the telomere to *EPA1*p). Silencing propagates from the telomere (T) and depends on the SIR complex (Sir2, Sir3, and Sir4) (2, 3, and 4), Rap1 (yellow circle), Rif1 (blue), and yKu proteins. *EPA3* expression responds also to osmotic stress and glucose starvation.

**Figure 2:**
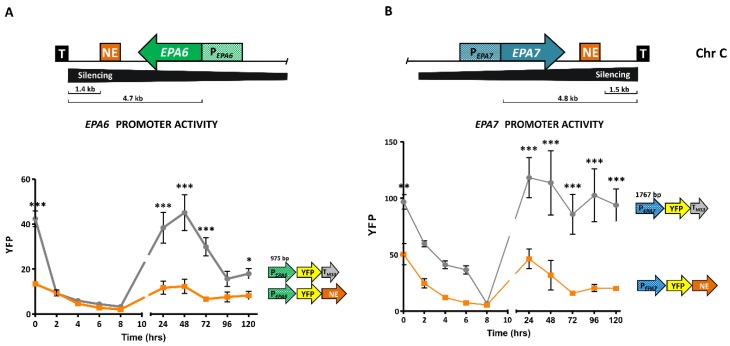
*EPA6* and *EPA7* promoters are negatively regulated by their respective negative elements (NEs). (**A top**) Schematic representation of the subtelomeric *EPA6* gene and its downstream NE. *EPA6* is repressed by subtelomeric silencing (indicated by a black horizontal triangle). (**A bottom**) Activity of the promoter of *EPA6* is negatively regulated by its NE. (**B top**) Schematic representation of the subtelomeric *EPA7* gene and its downstream NE. *EPA7* is repressed by subtelomeric silencing (indicated by a black horizontal triangle). (**B bottom**) Activity of the promoter of *EPA7* is negatively regulated by its NE. Replicative plasmids containing *EPA6* or *EPA7* promoter fused with YFP were transformed into *Candida glabrata* wild-type strain. One plasmid contained a heterologous 3′ UTR from the *HIS3* gene. A second plasmid contained the cognate *EPA6* or *EPA7* 3′ UTR containing its NE. Stationary phase cultures of each of these strains were diluted into fresh rich media and fluorescence was measured by FACS every two hours. Results are the mean of three biological repetitions. Statistical analysis was performed using 2 way ANOVA Bonferroni posttests. * means *p* < 0.05, ** *p* < 0.01 and *** *p* < 0.001.

*EPA1* is located close to the right telomere of chromosome E (Tel E_-R_), where it forms a cluster with *EPA2* and *EPA3* (Figure 1). Under most laboratory conditions, *EPA1* gene in the BG2 strain is not expressed, but it can be induced during the lag phase, immediately after a stationary phase culture is diluted into fresh media. Activity of the *EPA1* promoter is then negatively regulated by a cis-acting element, called the negative element (NE), which requires the yKu proteins and represses *EPA1* expression during logarithmic phase. There are nine additional copies of the NE in the CBS138 genomic sequence, some of which are associated to *EPA* genes, including *EPA6* and *EPA7*. We have shown that the expression of these genes is also negatively regulated by their associated NEs and independently of the vicinity of the corresponding telomere (Figure 2) [49]. Furthermore, *EPA1* expression is also negatively regulated by subtelomeric silencing, which is independent of the regulation of the NE [49]. In addition to these two mechanisms, this subtelomeric region contains a protosilencer, called Sil2126, localized between *EPA3* and the telomere, which helps propagate the heterochromatin assembled at the telomere repeats for up to 20 kb (Figure 1). Sil2126 has unique properties in that it is only active when placed close to its native telomere (Tel E_-R_), but inactive at similar distances from other telomeres [50]. The protosilencer Sil2126 depends on Rap1 and the SIR complex.

### 3.3. EPA1 Is Regulated by the Transcription Factor Pdr1

In the majority of the *C. glabrata* clinical isolates studied, Epa1 is the major adhesin conferring the ability to adhere to host cells in vitro and in vivo [9,11,12,41]. As described above, the transcriptional regulation of the *EPA1* gene is very complex [49,50,51,52,53]. More recent studies, using numerous different clinical isolates, some of which are isogenic strains isolated sequentially from the same patients after treatment with antifungal drugs (azoles/fluconazole), have revealed a very interesting connection between antifungal resistance and adherence with increased virulence. 

*C. glabrata* has unusually high rate of acquired resistance to azoles [51,52,53], and the major mechanism is through the induction of ABC-drug efflux pumps, especially Cdr1 and Pdh1. The regulation of transcription of these efflux pumps in *C. glabrata* requires the transcriptional activator Pdr1 [54,55]. Pdr1 needs to be activated by direct binding of xenobiotics such as fluconazole [56]. One of the most frequent mutations encountered in fluconazole resistant *C. glabrata* isolates or laboratory strains are gain of function mutations (GOF) in Pdr1. These mutant Pdr1 alleles do not require activation by fluconazole to induce transcription of not only efflux pumps, but also an array of stress response genes as well as genes involved in fatty acid metabolism, transcriptional regulation, and importantly, adhesion. Together these genes conform the pleiotropic drug resistance (PDR) network [41,53,54,57,58].

Analysis of two sequential (and related) clinical isolates taken 50 days apart from the same patient and after fluconazole treatment, led to the identification of a Pdr1 GOF allele (Pdr1^L280F^). By using isogenic strains carrying the wild-type Pdr1 allele or the GOF Pdr1^L280F^ it was demonstrated that this GOF allele confers resistance to fluconazole as well as increased adherence to epithelial cells. Furthermore, it was also found that the Pdr1^L280F^ allele resulted in diminished adhesion to, and uptake by, macrophages, although in a strain-specific manner [41]. Instead, the increased adherence to epithelial cells did not depend on the strain background. The GOF Pdr1^L280F^ allele led to an increased expression of *EPA1* mRNA and higher levels of adherence to epithelial cells. Indeed, other GOF alleles in Pdr1 also led to increased *EPA1* mRNA transcription and adherence, and these phenotypes are dependent on Epa1 [42]. 

## 4. Large Variability in the Pattern of Proteins Present at the Cell Wall among *C. glabrata* Clinical Isolates

### 4.1. Different C. glabrata Clinical Isolates Display a Unique Pattern of Proteins at the Cell Surface

Although several fluconazole resistant *C. glabrata* clinical isolates carrying GOF alleles in Pdr1 display Epa1-dependent increased adherence to epithelial cells, there are some notable exceptions like the clinical isolate DSY562. This isolate displays high levels of adherence to epithelial cells even though *EPA1* mRNA levels were very low compared to other clinical isolates [42]. Genome sequencing of DSY562 and its fluconazole resistant derivative carrying Pdr1^L280F^, allowed comparisons with the CBS138 genomic sequence. These studies demonstrated that while the two related DSY clinical isolates are very similar to each other, there are several major rearrangements when they are compared to CBS138. Most of the rearrangements involve CWP-encoding genes. It is tempting to speculate that under certain in vivo conditions nonallelic homologous recombination could be induced, and/or a DNA repair mechanism might be compromised. 

Genome sequencing and transcriptomic analysis of a different azole resistant clinical isolate (FFUL887) also showed that most of the genomic variation between this isolate and the CBS138 strain was found in CWP-encoding genes, especially in *PWP4*, *PWP5*, and *EPA8*. In addition, this study also showed that the expression of different adhesin-encoding genes varies with different Pdr1 GOF alleles [53]. Analysis of 4 pairs of clinical isolates, which were collected before and after azole treatment, led to the description of a set of Pdr1 GOF alleles conferring resistance to fluconazole but with a different impact on adherence to epithelial cells. In addition, the increased adherence was shown to be Epa1 dependent [9].

In another study, stationary phase cultures of two hyperadherent *C. glabrata* clinical isolates (PEU382 and PEU427) showed that *EPA* and *AWP* genes were not overexpressed compared to the CBS138 strain. The proteomic analysis led to the identification of 6 novel adhesins (Awp8-Awp13). These results show that there is a positive correlation between increased ability to adhere and incorporation of several adhesins at the cell wall [8]. 

*C. glabrata* is unique in that almost each clinical isolate analyzed has a different pattern of cell wall proteins present at the surface of the cell. In many cases, hyperadherent clinical isolates display overexpression of *EPA1*. However, there are other cases, like the ones described above, in which other adhesins at the cell wall could account for the high levels of adherence to host cells and plastic surfaces independently of Epa1. This could be advantageous since variants of the same initial strain will have different abilities to adhere to different surfaces during an infection where conditions could vary significantly and very rapidly.

In this regard, it is very interesting to point out that in some parasites like *Trypanosoma brucei* and *Plasmodium falciparum* as well as in another fungus (*Pneumocystis carinii*), families of adhesin-encoding genes that mediate cytoadhesion are localized at subtelomeric regions, and only one or very few of these genes are expressed at any given time. This facilitates the evasion of the immune system and allows for expansion of the adhesin-encoding gene family through nonallelic homologous recombination [36,59].

### 4.2. C. glabrata Shows Large Genomic Variability Involving CWP-Encoding Genes

The first report documenting large chromosomal rearrangements showed that there were many karyotypic changes among clinical isolates analyzed and some correlated with the appearance of resistance to fluconazole after clinical treatment [60]. More recent data indicates that indeed most of *C. glabrata* isolates from different sources display high genetic diversity [7,10]. This plasticity is clearly seen in adhesin-like encoding genes where more than 40% of these genes are deleted or duplicated [7]. For example, the subtelomeric *EPA* genes show increased copy number variation due to frequent duplication events. This was determined after sequencing sequential clinical isolates where *EPA2*, *EPA6*, *EPA8*, *EPA12*, *EPA22*, and some members of the *AWP* and *PWP* families were duplicated [10]. 

A systematic study of different CBS138 strains from different laboratories, has also shown large genomic rearrangements even under laboratory conditions where the cells are grown under non-stressful conditions [61]. Analysis of the regions of the genome where most of the rearrangements occurred showed that most of them were in CWP-encoding genes that contain tandem arrays of internal repeats. It is worth noting that some of the CBS138 laboratory strains showed differential expression of adhesins of the *EPA* and *AWP* families.

Lastly, the genomic sequence of an industrial strain of *C. glabrata* (CCTCC M202019) for pyruvate production showed that while its genome is very similar in general to that of the CBS138 strain, there were large differences in genes that are related to carbon metabolism and pyruvate secretion. Notoriously, CWP-encoding genes were also highly variable. The genomic analysis predicted 49 genes encoding typical adhesin-like genes of which only 6 were conserved between the industrial strain and CBS138. The other adhesin-like genes displayed variations in the number of the serine and threonine-rich repeats, of which deletions were the most common. The authors also showed that this industrial strain is less adherent to endothelial cells in vitro [62]. 

In summary, these studies highlight the great genomic variability between different *C. glabrata* isolates with regard to the adhesins expressed at the cell surface and the large tolerance that *C. glabrata* displays to these gross rearrangements. It is not known whether the rearrangements and mutations that lead to genomic plasticity are stochastic or induced by environmental cues. It is possible that the efficient generation of genomic variability has been selected as a pathway to respond to harsh conditions to acquire traits that confer increased adherence to a variety of cells and surfaces, increased resistance to the presence of xenobiotics and/or to evade the immune system [12,63]. Indeed the genomic plasticity that results in a wide variety of gene expression patterns probably insures the success of *C. glabrata* as a pathogen. This raises the issue that generalizations about the behavior of *C. glabrata*, cannot be made based in only one strain.

## 5. Role of Epa1 in Host Cell Recognition of *C. glabrata*

### Interactions with Cells from the Immune System: Neutrophils, Macrophages, Monocytes, Natural Killers (NK), and Dendritic Cells

Cells of the innate immune system, such as natural killers (NK), neutrophils, macrophages (derived from monocytes), and dendritic cells are the first line of defense against *Candida* infections. *C. glabrata* has evolved mechanisms that allow it to survive the attack of macrophages, and even replicate within them [64,65]. For phagocytosis to occur, *C. glabrata* must make an initial contact with the phagocytic cell. This contact is mediated by surface receptors in the immune cells and recognition molecules exposed on the cell wall of the fungus. Immune cells have pattern-recognition receptors (PRR), such as Toll-like receptors (TLRs), Dectin receptors (CLR) Dectin-1 and Dectin-2, mannose receptor (MR), and galectin-3, among others (Table 1) [66]. On the fungal side of this interaction, the cell wall of *C. glabrata* is a complex structure composed of two layers. The inner layer is a network of carbohydrates (β1,3 and β1,6-glucans and chitin) and the outer layer is constituted mainly of glycosylated mannoproteins, which are covalently bound to the inner layer glucans [67]. Adhesins in *C. glabrata* form part of the mannoproteins at the cell wall. The CLR receptors Dectin-1 and Dectin-2 recognize β-glucans and mannans on the surface of the fungus and in this way trigger the immune response against *Candida*. Dectin-1 binds β-glucans inducing phagocytosis and the respiratory burst [68], release of neutrophil extracellular traps or NETs [69], and cytokine production [70]. Dectin-2 was first described to specifically recognize mannans on the hyphae in *C. albicans* [71], and together with Dectin-1 triggers a Th1 response against *C. albicans* [72]. However, in a later study using Dectin-2^−/−^ knockout mice, it was shown that neutrophils from these mice were less efficient at internalizing *C. glabrata* cells and triggered a weaker oxidative burst than neutrophils from wild type mice. This indicated that Dectin-2 receptors are important for *C. glabrata* recognition and not just for *C. albicans* hyphae recognition [73].

The interaction between Dectin-1 and β-glucans determines *C. glabrata* colonization in a murine model of infection and importantly, the degree of β-glucans exposure at the cell surface is directly related with the fitness of *C. glabrata* in the gastrointestinal tract [74]. Thus, Dectin-1 plays a predominant role in the response against *C. glabrata* while Dectin-2 depends strongly on the fungal burden [75]. However, the specific ligand on *C. glabrata* recognized by Dectin receptors has not been identified yet.

It has been shown that Epa1 mediates interaction between *C. glabrata* and human macrophages. Heterologously expressed *CgEPA1* in the nonadherent *S. cerevisiae* resulted in adherence of *S. cerevisiae*-p*EPA1* to human derived macrophages. Adherence is followed by phagocytosis and cytokine release (IL-8 and TNF-α), however, *C. glabrata* cells expressing *EPA1* adhere efficiently to human macrophages but phagocytosis and cytokine release are inhibited. It is important to point out that Epa1 expressed in *S. cerevisiae* can also mediate adherence to murine derived macrophages, but only after Dectin-1 receptors have been blocked. This highlights the important differences between murine and human derived macrophages [17]. 

NK cells are a different type of lymphocytic cell from the innate immune system that can recognize cells that have been infected by viruses, bacteria, or tumoral cells through different cell surface receptors. NK cells are important to fight fungal infections. Human NK cells are capable of recognizing *C. glabrata* cells through the NKp46 receptor and also by murine NK cells through the orthologous murine receptor NCR1. The NKp46 receptor specifically recognizes Epa1, Epa6, and Epa7. Clearance of *C. glabrata* systemic infections in mice depends on the presence of these adhesins and the NCR1 receptor on the murine NK cells [18]. It is proposed that NKp46/NCR1 may be a new type of PRR in which the receptor is glycosylated with the same pattern as the host cells. *C. glabrata* cells then recognize the glycosylation pattern of the NKp46/NCR1 through Epa1, Epa6, and Epa7 and bind to the NK cells. In this way, *C. glabrata* is lured to the NK cells and killed [18].

In addition to adherence of *C. glabrata* to NK cells and macrophages, there is evidence that shows that *C. glabrata* can activate murine bone marrow-derived dendritic cells through the toll-like receptor TLR7 and resulting in the release of IFN-β. This modulates the immune response through IFNs-I signaling [16].

## 6. Interactions with Other Microorganisms

In addition to *C. glabrata* interactions with host cells, it is well known that *C. glabrata* can interact efficiently with other microorganisms. Early studies found that a sizable proportion of candidemias are actually mixed infections. Up to 24% of the reviewed cases presented concomitant bacteremia and between 3% and 4% of the candidemias contained more than one *Candida* species. Interactions between *C. glabrata* with *C. albicans*, *C. krusei*, and *C. tropicalis* have also been detected [81]. In terms of clinical consequences, mixed infections are difficult to diagnose and treat because they sometimes need changes in treatment [81] and in some cases have been associated with higher mortality rates [87]. Initially, it was discovered that *C. albicans* requires Als1, Als3, and Als5 for cell-to-cell self-aggregation. In contrast, *C. glabrata* does not form aggregates. However, when *C. glabrata* and *C. albicans* are mixed, cells can aggregate suggesting that the interaction between *C. albicans* and *C. glabrata* might be important for pathogenesis of *C. glabrata* [88]. *C. glabrata* by itself is rarely found in oral candidiasis, but it is more frequently found in combination with *C. albicans*. In studies performed in reconstituted human oral epithelium, it was found that for *C. glabrata* single infections, colonization of the oral epithelium was strain dependent. However, *C. albicans* was able to increase invasiveness of all the *C. glabrata* strains tested, including those that were less efficient at colonization [89]. 

*C. glabrata* and *C. albicans* can form mixed biofilms because *C. glabrata* adheres tightly to *C. albicans* hyphae (not to the yeast form). *C*. *glabrata* recognizes the hypha-specific adhesins Als1 and Als3. Furthermore, heterologous expression of *ALS1* and *ALS3* in *S. cerevisiae* prompted adherence of *C. glabrata* to Als1 and Als3 expressing *S. cerevisiae* cells. Importantly, coinfection of *C. albicans* and *C. glabrata* in a mouse model of oropharyngeal infection (OPC), showed that when *C. albicans* is present invading the oral epithelium, it allows for colonization and invasion of *C. glabrata* in proportion to the level of *C. albicans* invasion [79]. In this interaction, the *C. albicans* adhesins Als1 and Als3 and the *C. glabrata* Epa8, Epa19, Awp2, and Awp7 adhesins and the CAGLOF00181g adhesin-encoding gene are thought to play an important role [79]. Consistently, with the differential expression of adhesin-encoding genes discussed in this review, different *C. glabrata* strains displayed different levels of adherence to *C. albicans* hyphae [79].

*C. albicans* and *C. glabrata* mixed infections are also commonly found in vaginal infections. In a study using reconstituted human vaginal epithelium, it was found that even though *C. glabrata* alone can colonize the vaginal epithelium, the presence of *C. albicans* resulted in increased colonization, invasiveness, and tissue damage. This suggests that *C. albicans* could be improving the invasiveness of *C. glabrata* in this in vitro model [43]. Besides mixed *Candida* infections, there is a report of an invasive infection of *C. glabrata* with *Aspergillus fumigatus*, which was resistant to monotherapy antifungal treatment that had to be modified [85].

There are some case studies that have found mixed infections of *C. glabrata* and bacteria, for example *C. glabrata* with *Serratia marcescens*. This mixed infection was associated with recurrent *C. glabrata* infections at the particular site [86]. *C. glabrata* has also been found together with *Pseudomonas aeruginosa*, and in this case *C. glabrata* biofilms were inhibited by the presence of *P. aeruginosa* [84].

## 7. Conclusions

The ability to adhere to different host cell types, abiotic surfaces such as medical devices, and other microorganisms is thought to be an essential first step in *Candida glabrata* pathogenesis. In this regard, *C. glabrata* has evolved to acquire an unusually large number of genes encoding cell wall proteins (CWPs). In the pathogenic species within the *Nakaseomyces* genus, the appearance of the ability to colonize the mammalian host coincides with the large expansion of adhesin-like encoding gene families, specifically the *EPA* gene family.

An extremely relevant characteristic of *C. glabrata* is that there is a large genomic variability among clinical isolates and even between cultures of the same standard strain CBS138 from different laboratories. Importantly, the largest variation occurs within the CWP-encoding genes. This has a huge impact on the variability that can be generated in CWPs. As a consequence every strain analyzed seems to display a unique profile of cell surface proteins and this has a direct impact on the ability to adhere to different types of host cells, to abiotic surfaces, to other pathogens, or avoid the immune system. 

In addition, each of these genes is tightly regulated by several layers of regulation. This in turn depends on the particular strain. Subtelomeric silencing provides a global regulatory network, but in addition there are *cis*-acting elements, transcriptional activators, and repressors that could be sensing nutritional signals, oxidative stress, and the presence of xenobiotics, among others.

Thus, pathogenicity and ability to adhere to many cell types, including other microorganisms and abiotic surfaces, and the ability to respond to xenobiotics are tightly correlated in *C. glabrata*.

Future characterization of sequential clinical isolates that include transcriptomic and proteomic analysis and correlation with clinical treatment will help understand the cues to which *C. glabrata* responds to in the host and how it is manifested in the cell wall composition. Another important issue to understand is whether *C. glabrata* has evolved to respond to stressful conditions by triggering high rates of homologous recombination. This would generate genomic variability in the population and thus insure that some individuals will survive.

A detailed understanding of the evolution of adhesin-encoding genes and the regulation of their expression is of pivotal importance to host–microbe interactions to devise novel treatments for these serious infections.

## Figures and Tables

**Table 1:** Summary of the types of cells to which *Candida glabrata* can adhere in vitro and in vivo, the receptors in host cells, microorganisms, or substrates involved in the interaction, and the ligand on *C. glabrata* recognized by the receptor.

Interaction with		Receptor in the Host or Microorganism ^b^	Ligand on *C. glabrata* ^d^	Reference
Type of Cells	Specific Cell Line ^a^			
Epithelial cells	HEp2 (human), CHO-Lec2 (hamster)	ND ^c^	Epa1	[11]
	Cardiac endothelium (guinea pig)	ND	ND	[15]
	Caco-2 (human)	ND	Epa1	[29]
	Lec2 (hamster)	ND	Epa6, Epa7	[33]
	OKF6/TERT-2 (human)	ND	β-glucan	[76]
	UVECS (human)	ND	Pwp7, Aed1	[14]
	OKF6/TERT-2 (human)	CDw17	ND	[77]
Immune cells	Natural Killers (NK)	Nkp46 (human) NCR1 (murine)	Epa1, Epa6, Epa7	[18]
	Macrophages (human/murine)	Dectin1, Dectin2	β-glucan	[73,74,75]
		ND	Epa1	[17]
	Dendritic Cells (murine)	ND	Epa1/ND	[16,17]
	Neutrophils (murine)	Dectin2	β-glucan	[73]
Platelets (murine)		ND	ND	[78]
*Candida* spp.	*C. albicans* (hyphae)	Hwp1, Als3, Als1	Epa8, Epa19, Awp2, Awp7, CAGL0F00181	[79]
	*C. krusei*	ND	ND	[80,81]
Others	Fibronectin		Epa6	[82]
	Osteoblast (human)	ND	ND	[83]
	*S. marscesens*, *P. aeruginosa*, *A. fumigatus*	NI ^e^	NI	[84,85,86]

^a^ Name of the cell lines or types of immune cells used to determine adherence. Abbreviations mean: HEp2 = Human Epidermoid cancer cells. CHO = Chinese Hamster Ovary. Lec2 = CHO derived mutants in CMP-syalic acid translocation. Caco-2 = adeno Carcinoma of the colon. OKF6/TERT-2 = human Oral Keratinocytes expressing hTERT subunit. UVEC = Umbilical Vein Endothelial Cells. The origin of the cell type (human or murine) is indicated in parenthesis. ^b^ Receptor identified on the surface of the cell to which *C. glabrata* adheres. ^c^ ND means not determined. ^d^ Ligand identified on *C. glabrata* that is recognized by the receptor. ^e^ NI means not investigated.
